# Supplementary material for: Interpolymer Complexes of Eudragit® Copolymers as Novel Carriers for Colon-Specific Drug Delivery
Source: Polymers (Basel). 2020 Jun 30;12(7):1459. doi: 10.3390/polym12071459 (PMC7407155; doi:10.3390/polym12071459)
Supplement: Supplementary file 1 [file polymers-12-01459-s001.pdf]

Supporting information

Interpolymer complexes of Eudragit® copolymers as novel carriers for colon-specific drug delivery

Aleksandra V. Bukhovets [1], Nikoletta Fotaki [2], Vitaliy V. Khutoryanskiy [1,3] and Rouslan I. Moustafine [1,*]

[1] Institute of Pharmacy, Kazan State Medical University, 16 Fatykh Amirkhan Street, 420012 Kazan, Russian Federation; a.bukhovets@gmail.com (A.V.B.) and rouslan.moustafine@gmail.com (R.I.M)
[2] Department of Pharmacy and Pharmacology, University of Bath, Claverton Down, Bath, BA2 7AY, United Kingdom; n.fotaki@bath.ac.uk (N.F.)
[3] Reading School of Pharmacy, University of Reading, Whiteknights, PO box 224, Reading RG66AD, United Kingdom; v.khutoryanskiy@reading.ac.uk (V.V.K.)

Table 1S. Composition of biorelevant media taken from [1, 2].

FaSSGF		FaSSIF-V2		FaSSCoF	
Sodium taurocholate	80 µM	Sodium taurocholate	3 mM	Tris(hydroxymethyl)-aminomethane	5.5 g
Lecithin	20 µM	Lecithin	0.2 mM	Maleic acid	8.8 g
Pepsin	0.1 mg/mL	Blank buffer:		Bile salt extract	0.113 g
Sodium chloride	34.2 mM	Maleic acid	19.12 mM	Lecithin	0.222 g
Hydrochloric acid to achieve specific pH	pH 1.6	Sodium hydroxide	34.8 mM	Palmitic acid	0.026 g
Deionized water (up to)	1 L	Sodium chloride	68.62 mM	Bovine serum albumin	3.0 g
		Blank buffer to achieve specific pH	pH 6.5	Sodium hydroxide to achieve specific pH	pH 7.8
		Deionized water (up to)	1 L	Deionized water (up to)	1 L

Table 2S. Conditions of dissolution tests in buffer solutions (USP Apparatus III and IV).

pH	Period from beginning of the experiment, min	Flow rate (mL/min) / Dip rate (dpm)
1.2	0-60	16/5
5.8	60-180	16/5
6.8	180-300	16/5
7.4	300-420	16/5

Table 3S. Conditions of dissolution tests in biorelevant media (USP Apparatus III and IV).

Medium	Period from beginning of experiment, min	Flow rate (mL/min) / Dip rate (dpm)
FaSSGF	0-60	8/5
FaSSIF-V2	60-270	4/5
FaSSCoF	270-420	4/5

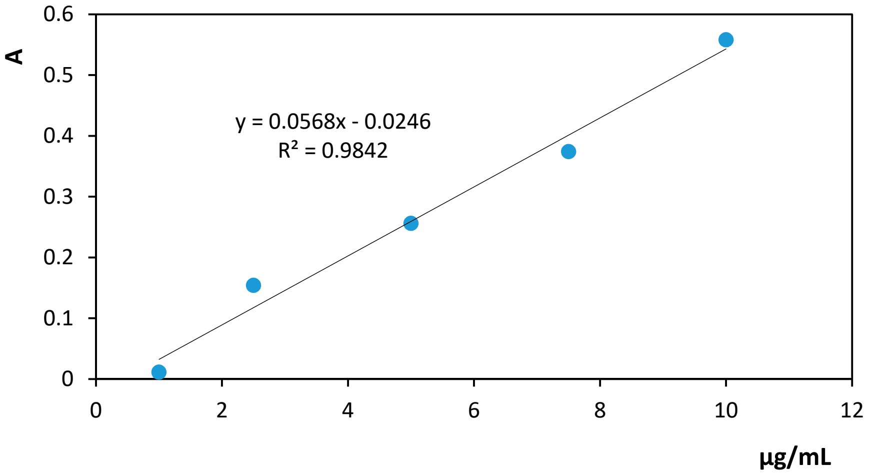

(**a**)

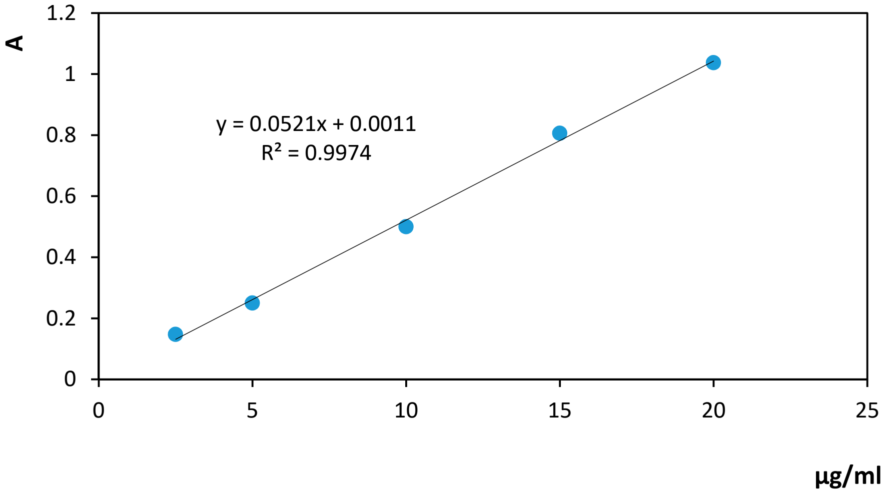

(**b**)

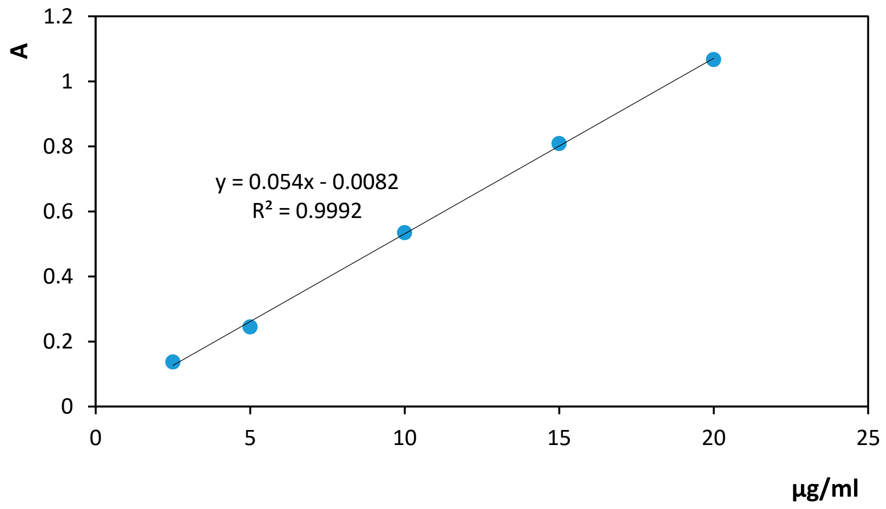

(**c**)

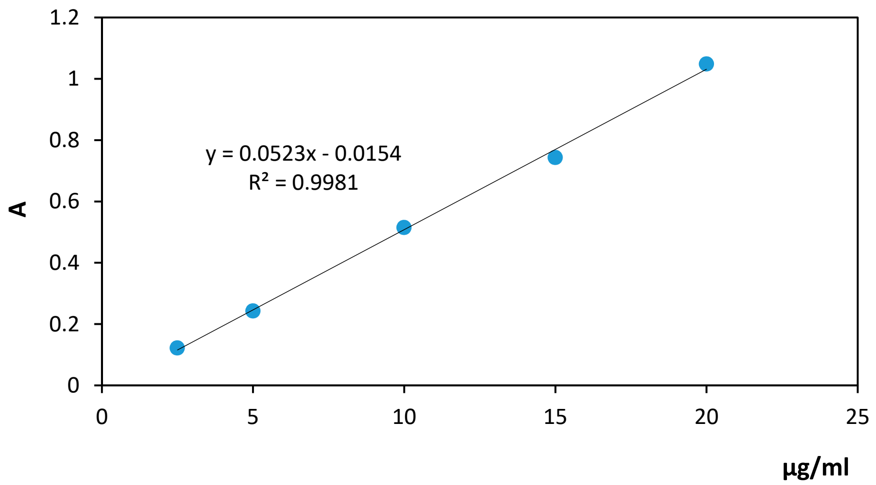

(**d**)

Figure 1S: Calibration curves of indomethacin in buffer solutions: (**a**) pH 1.2; (**b**) pH 5.8; (**c**) pH 6.8; (**d**) pH 7.4.

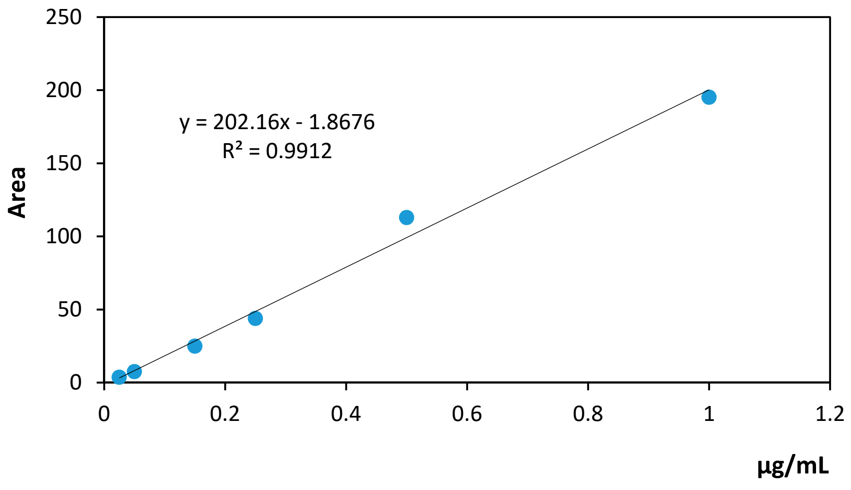

(**a**)

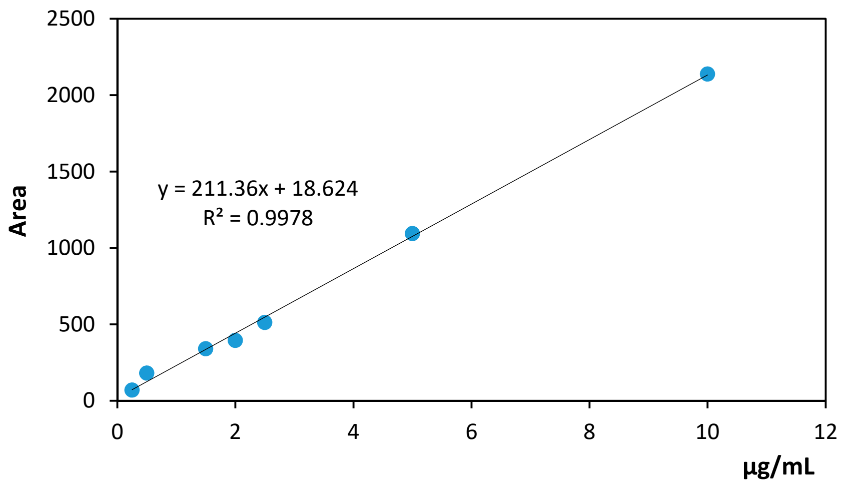

(**b**)

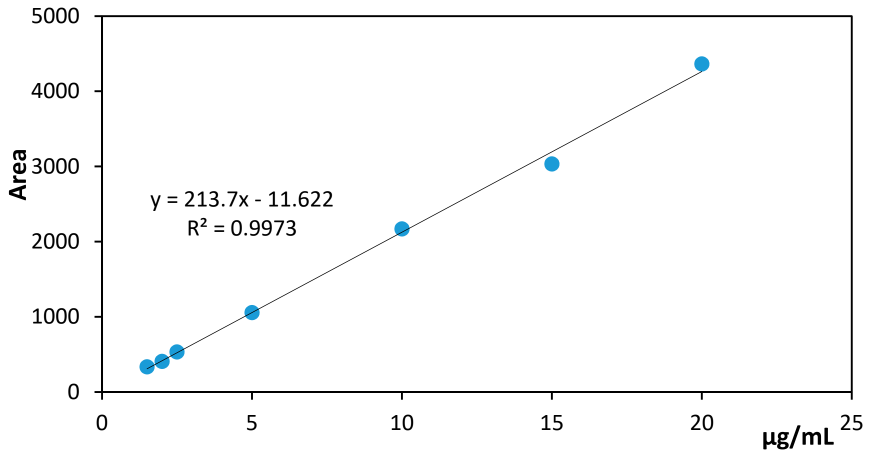

(**c**)

Figure 2S: Calibration curves of indomethacin in biorelevant media: (**a**) FaSSGF; (**b**) FaSSIF-V2; (**c**) FaSSCoF.

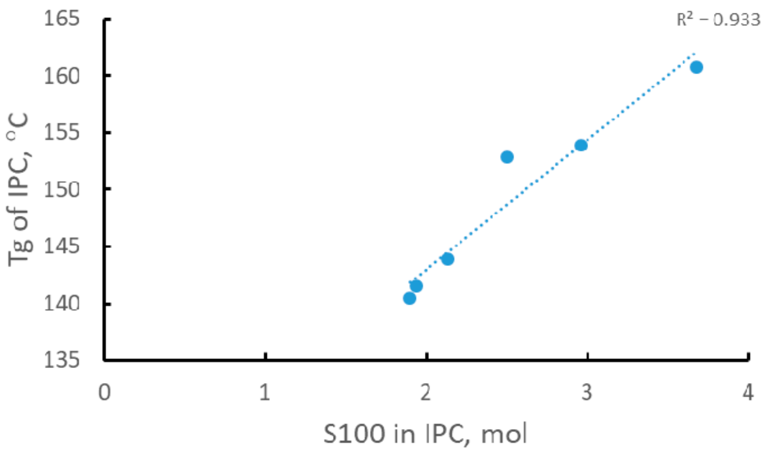

Figure 3S. Dependence of Tg on the content of S100 in IPC

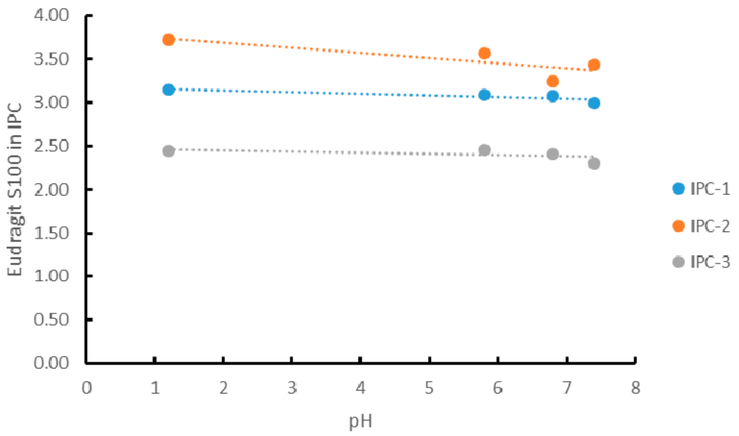

Figure 4S. Changes in the composition of interpolymer complexes in buffer solutions mimicking the pH values of different parts of gastrointestinal tract

References

1. Jantraid, E.; Janssen, N.; Reppas, Ch.; Dressman, J.B. Dissolution Media Simulating Conditions in the Proximal Human Gastrointestinal Tract: An Update. *Pharm. Res*. **2008**, *25*: 1663. https://doi.org/10.1007/s11095-008-9569-4
2. Vertzoni, M.; Diakidou, A.; Chatzilias, M.; Söderlind, E.; Abrahamsson, B.; Dressman, J.B.; Reppas, Ch. Biorelevant Media to Simulate Fluids in the Ascending Colon of Humans and Their Usefulness in Predicting Intracolonic Drug Solubility. *Pharm. Res*. **2010**, *27*, 2187-2196. https://doi.org/10.1007/s11095-010-0223-6
